# 

**Published:** 1893-04-29

**Authors:** 


PRICE TWOPENCE.

No. 344, Vol. XIV.—April 29th, 1893.

[Registered at the General Post Office as a Newspaper for transmission in the United Kingdom and Abroad.]

WITH EXTRA NURSING SUPPLEMENT

Per Annum, 8s. 6d., post free.

Monthly Parts, 6d.; post free, 8d.

Ditto per Annum, 8s., post free.

### THE HOSPITAL

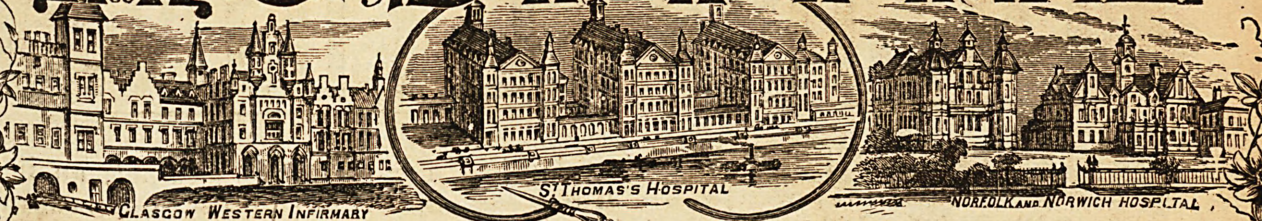

WITH EXTRA NURSING SUPPLEMENT.

No. 344. Vol. XIV.] SATURDAY, APRIL 29TH, 1893. [TWOPENCE.

